# Autophagy in Neurotrauma: Good, Bad, or Dysregulated

**DOI:** 10.3390/cells8070693

**Published:** 2019-07-10

**Authors:** Junfang Wu, Marta M. Lipinski

**Affiliations:** 1 Department of Anesthesiology and Center for Shock, Trauma and Anesthesiology Research (STAR), University of Maryland School of Medicine, Baltimore, MD 21201, USA; 2 Department of Anatomy and Neurobiology, University of Maryland School of Medicine, Baltimore, MD 21201, USA; 3 Center to Advance Chronic Pain Research, University of Maryland, Baltimore, MD 21201, USA

**Keywords:** spinal cord injury, traumatic brain injury, autophagy, autophagic flux, neuronal cell death, lysosomal damage

## Abstract

Autophagy is a physiological process that helps maintain a balance between the manufacture of cellular components and breakdown of damaged organelles and other toxic cellular constituents. Changes in autophagic markers are readily detectable in the spinal cord and brain following neurotrauma, including traumatic spinal cord and brain injury (SCI/TBI). However, the role of autophagy in neurotrauma remains less clear. Whether autophagy is good or bad is under debate, with strong support for both a beneficial and detrimental role for autophagy in experimental models of neurotrauma. Emerging data suggest that autophagic flux, a measure of autophagic degradation activity, is impaired in injured central nervous systems (CNS), and interventions that stimulate autophagic flux may provide neuroprotection in SCI/TBI models. Recent data demonstrating that neurotrauma can cause lysosomal membrane damage resulting in pathological autophagosome accumulation in the spinal cord and brain further supports the idea that the impairment of the autophagy–lysosome pathway may be a part of secondary injury processes of SCI/TBI. Here, we review experimental work on the complex and varied responses of autophagy in terms of both the beneficial and detrimental effects in SCI and TBI models. We also discuss the existing and developing therapeutic options aimed at reducing the disruption of autophagy to protect the CNS after injuries.

## 1. Introduction

The role of autophagy after the central nervous system (CNS) insults is under perusal, as investigation has begun to determine how autophagy and related pathways contribute to secondary injury and functional recovery following neurotrauma [1,2,3,4,5], including spinal cord injury (SCI) and traumatic brain injury (TBI). Whether detected autophagic responses to injury are protective or detrimental remains controversial, with experimental reports revealing protective effects of both enhancing and hindering autophagy [1,6].

Autophagy is a vital intracellular degradation pathway that delivers cytoplasmic constituents to the lysosomes for degradation [7]. There are three different types of autophagy that have been descripted so far, including macroautophagy, microautophagy, and chaperone-mediated autophagy [8,9,10]. In addition, several types of targeted autophagy have been described, including mitophagy, which is a selective form of macroautophagy that specifically targets and degrades damaged mitochondria. Macroautophagy involves the formation of a cytoplasmic membrane that engulfs cargo and eventually elongates to form double-membrane vesicles termed autophagosomes. It culminates in the fusion of cargo-containing autophagosomes to the lysosomes and the degradation of the cargo by lysosomal hydrolases [11]. As macroautophagy is the best characterized form of autophagy, hereafter, the term autophagy refers to macroautophagy unless otherwise specified. Under normal conditions, autophagy is an essential physiological process that maintains a balance between the manufacture of cellular components and breakdown of damaged organelles and other toxic cellular constituents. Its processing is strictly regulated under physiological conditions [12]. Recent considerable progress has demonstrated that autophagy is dysregulated in injured CNS following trauma, and may play either beneficial or detrimental functions after injury, depending on the context and the mechanisms leading to its perturbation [1,6,13,14]. Emerging data also suggest that the restoration and/or augmentation of proper autophagy function, for example by inducing lysosomal biogenesis, may be a potential therapeutic target for TBI and SCI.

This review explores the current research on the function and mechanisms of autophagy in two models of CNS injury: SCI and TBI. Cell-type specific responses of autophagy are discussed according to different locations, severity, and time windows of traumatic injury. We discuss recent findings suggesting that impairment of the autophagy–lysosomal pathway may be part of the secondary injury processes of SCI/TBI. We also review recent studies and novel mechanistic discoveries on cytosolic phospholipase A2 (cPLA2) participation in lysosomal damage, and provide therapeutic options, with an emphasis on the pharmacological modulation of autophagy and lysosomal biogenesis for neuroprotection and the prevention of neuroinflammation after CNS trauma.

## 2. Traumatic Spinal Cord and Brain Injury and Their Injury Mechanisms

According to the National Spinal Cord Injury Statistical Center [NSCISC, 2018], in the United States (U.S.), there are approximately 288,000 people living with SCI, and 17,000 new SCI cases occur each year. The impact of these injuries is devastating for individuals, and the health care costs associated with the injury are some of the highest in the U.S. [15,16]. Experimental and clinical studies [17,18,19,20,21] have indicated that acute SCI is a two-step process involving primary and secondary mechanisms. Primary injury of the spinal cord refers to the initial mechanical tissue damage. It also includes ruptured microvessels in the spinal cord, and subsequent hemorrhage and edema that are associated with secondary injury. SCI-mediated secondary injury processes include a cascade of biochemical and cellular events [22], such as free-radical formation, excitatory amino acid release, axonal damage, neuronal and oligodendroglial cell death, the infiltration of macrophages and peripheral immune cells, microglia, and astrocyte activation/glial scar formation. These delayed secondary injury processes can occur over hours, days, and months after initial impact [23,24], contributing to progressive neuronal degeneration and neurological dysfunction. Despite considerable research over the past 30 years, there is still no established effective treatment to improve recovery following SCI. In part, this reflects an incomplete understanding of the complex secondary pathobiological mechanisms involved. 

TBI represents a major public health problem, with more than 1.7 million new cases annually in the United States [25] and accounting for 60% of all trauma deaths in the U.S. [26]. Similar to SCI, TBI causes cell loss and neurological functional deficits through both the direct physical damage of tissue (primary injury), and through subsequent biochemical changes (secondary injury). The latter reflects delayed and potentially reversible molecular and cellular pathophysiological mechanisms [27,28], which begin within seconds to minutes after the primary insult, and may continue for months to years [29,30]. Such secondary injury processes, similar in SCI, lead to cellular changes that include neuronal and oligodendroglial cell death, as well as microglia and astrocyte activation and glial scar formation. Although most of the research to date has been directed at early cellular and molecular events, both experimental and clinical evidence suggests that CNS trauma-mediated pathophysiological changes may continue for years [30,31,32,33], leading to chronic post-mitotic cell loss and microglial/astrocytes activation, and contributing to chronic functional deficits.

Cell death after neurotrauma is a major cause of neurological deficits and mortality. Although CNS injury induces changes in multiple cell types such as neurons and oligodendrocytes, the mechanisms of neuronal cell death have been the predominant focus. There are multiple cell death mechanisms in injured CNS following trauma [34,35]. Three main morphological types of cell death include apoptosis, autophagic cell death, and necrosis. Molecular and biochemical pathways that are involved in cell death after both TBI and SCI include, among others, caspases-dependent apoptosis (the Bcl-2 family), cell cycle activation-dependent pathways, the autophagy-related (ATG) protein family-mediated cell death (autophagy), caspase-independent programmed cell death [including extracellular-signal-regulated kinase 2 (ERK2), poly[Adenosine diphosphate (ADP)-ribose] polymerase (PARP)-1, apoptosis-inducing factor (AIF)], calpains and cathepsis-mediated calcium-dependent cell death, and the c-Jun N-terminal kinase-mediated non-apoptotic cell death (oncosis) [34,36,37]. Interestingly, apoptosis and autophagy share many regulatory factors, including the Bcl-2 family proteins [38]. The pro-apoptotic B-cell lymphoma 2 (Bcl-2) proteins Bim, Bid, and Bax, are known mediators of lysosomal membrane permeabilization (LMP) [39,40,41,42], while the anti-apoptotic Bcl-1 and Mcl-1 negatively regulate autophagy through interaction with Beclin 1. Additionally, necroptosis, the regulated receptor interacting protein kinase (RIPK)-dependent necrosis pathway, may also contribute to secondary neuronal cell death after CNS insults [3,43,44]. Obstacles to successful therapy against neurotrauma-induced neuronal cell death include the diversity of cell death pathways, which have both overlapping and distinct molecular mechanisms, and the narrow therapeutic windows for some types of neuronal cell death [34,45]. Thus, we emphasize that the effective neuroprotective strategies will need to concurrently modulate multiple signaling pathways to reflect the spatial and temporal changes underlying the diversity of neuronal cell death.

Stimulation by pro-inflammatory cytokines, chemokines, or alterations in the CNS environment induces microglial activation and the attraction of macrophages to the injury site [46,47]. Activated microglia undergo a transition from a resting, ramified phenotype to a phagocytosis-capable, ‘macrophage-like’ phenotype that is virtually indistinguishable from blood-borne macrophages [48,49,50]. Microglia and macrophages are found in the injured CNS within 12 to 24 h post-injury, with maximal concentration at four to eight days post-injury [49,51]. These cells produce free radicals, nitric oxide, and arachidonic acid derivatives, as well as a number of cytokines and chemokines in an effort to remove debris and dysfunctional cells. However, excessive inflammation may also result in the death of neighboring undamaged cells [52,53,54,55,56]. It is reported [57] that the activation of microglia-associated inflammatory factors may continue indefinitely after CNS trauma: certainly, it lasts for many months in rodents.

## 3. Autophagy, Autophagy Flux, and the Lysosomal Functions in Neurotrauma

### 3.1. Autophagy and Autophagy Flux

The process of autophagy includes several essential steps, including the induction, sequestration of cargo within the autophagosomal membrane, maturation of the autophagosomes, and degradation [12,58]. While baseline autophagy proceeds at all times, in adverse conditions such as environmental stress, nutrient starvation, or acute cellular injury, it is further activated. Cytoplasmic components including damaged organelles and toxic protein aggregates are sequestered by a unique membrane called the phagophore or isolation membrane. Complete sequestration by the elongating phagophore results in the formation of the autophagosome, which is typically a double-membraned vesicle. Then, autophagosomes are transported within the cell and fuse with lysosomes. Then, the inner membrane of the autophagosome and the cytoplasm-derived materials contained in the autophagosome are degraded by lysosomal hydrolases. The whole process of autophagy is referred to autophagic flux [7], which represents the dynamic process of autophagy from cargo sequestration to its degradation. 

Completion of the autophagic flux requires the coordinated activity of various members of the autophagy-related (ATG) protein family [59,60]. For example, ATG13, ATG101, and FIP200 (homolog of yeast ATG17) are part of the ULK1 kinase complex that initiates autophagy. ATG9 also mediates an essential function in early autophagosome formation. Expansion of the autophagosomal membrane depends on two ubiquitin-like conjugating systems: the ATG12–ATG5–ATG16L system and the phosphatidylethanolamine (PE)–LC3 system. LC3 is the commonly used name for microtubule-associated protein 1 light chain 3β (MAP1LC3B). Upon activation of the autophagic pathway, the cytosolic, proteolytically processed form of LC3 (termed LC3-I) is lipidated to form LC3–PE (termed LC3-II), which is specifically recruited to the phagophore membrane. LC3-II accumulates on autophagosomal membranes and—through interaction with adaptor proteins—helps recruit substrates that are tagged for autophagic degradation. Thus, LC-II levels correlate well with autophagosome numbers. In addition, rapamycin complex 1 (MTORC1) operates as a central suppressor of autophagy. Conversely, beclin 1 (BECN1), a regulatory subunit of the type III phosphatidylinositol (PI) 3 kinase, promotes autophagy by promoting the formation of phosphatidylinositol 3 phosphate, which is necessary for autophagosome formation [60]. Moreover, ubiquitylated structures, including protein aggregated and in some cases mitochondria, are bound by the autophagic adaptor sequestosome 1 (SQSTM1; also known as p62), which enables their uptake by autophagosomes through LC3-II [61]. p62/SQSTM1 is one of several factors that target specific cargoes for autophagy, also including NBR1 [neighbor of breast cancer type 1 (BRCA1) gene], NDP52 (nuclear domain 10 protein 52), and OPTN (optineurin).

A number of methods are currently utilized to assess autophagic flux [62]. Transmission electron microscopy can be used for the partial analysis of the autophagosome pool size in vitro and in vivo without a time dimension. One of the most common ways to monitor autophagy is by measuring LC3-II protein turnover, which is incorporated into autophagosomes and then degraded in the lysosome. There are many different ways to measure LC3-II protein levels, including Western blot and immunofluorescent microscopy. However, these methods are complicated by the LC3-II levels increasing with autophagy induction due to increased autophagosomal formation, but also decreasing as these autophagosomes are turned over. Thus, a more reliable way to accurately monitor LC3-II levels is with a flux assay that uncouples autophagosome formation from its degradation by using lysosomal inhibitors such as bafilomycin-A1 (V-ATPase inhibitor) or chloroquine (a lysosomotropic compound that neutralizes lysosomal pH). As the flux is a rate, an analysis of both LC3-II and p62 levels over time in the presence and absence of lysosomal inhibitor is required. Another possibility in vitro is monitoring the half-life of labeled long-lived proteins that are known to be degraded via autophagy [63]. Fluorescence microscopy is an extremely valuable tool for the assessment of autophagic flux and the autophagosome pool size per cell. Use of the green fluorescent protein (GFP)–LC3 reporter allowing direct visualization of autophagosomes has been especially valuable. It is widely used for the evaluation of autophagy flux in vitro via image-based flux assay and live cell microscopy, and of the autophagosome pool in vivo using transgenic *GFP-LC3* reporter mice. Recent developments of the dual RFP-GFP-LC3 reporter further improved the evaluation of autophagy flux. It takes advantage of differential pH sensitivity of GFP (acid labile) and RFP (acid stable) to directly assess the fusion of autophagosomes (red and green) with lysosomes to form autolysosomes (red only) [62]. However, it remains challenging to employ these techniques in vivo in a manner that uniformly expresses autophagic flux quantitatively and assesses the magnitude of change in flux.

### 3.2. Impairment of Autophagy Flux in SCI and TBI

Increased markers of autophagy have been observed in different experimental SCI models [64,65,66,67,68]. Several reports indicate that the accumulation of autophagosomes is initiated very early during secondary injury, in some cases within hours after the initial impact. In SCI, depending on the injury models and severities as well as different species and sex, some reported significant increases in expression levels of the autophagosome marker LC3-II during the first 24 h after SCI [3,4,67,69,70], whereas others have only detected a delayed increase of this marker three to seven days after injury [64,65,68,71,72,73]. In some cases, the expression of Beclin 1, a key protein in the initiation of autophagy [74], has been reported to increase after SCI, supporting the hypothesis that SCI may lead to an increase of autophagy initiation [64,65,73,75,76]. However, other works have reported unaltered levels of Beclin 1 and other upstream mediators and regulators of autophagy [4,68], suggesting that increased autophagy initiation is unlikely to account for an increase in the number of autophagosomes. The p62/SQSTM1 protein, similar to all autophagy cargo adaptors, is degraded by autophagy along with its cargo. Therefore, provided no changes in transcription/synthesis are observed, p62 levels can be often used to approximate the autophagy-dependent degradation rate, with low levels of p62 protein associated with high autophagy flux (high degradation) and high p62 levels associated with low autophagy flux (inhibited degradation). Most reports describe the accumulation of both LC3 and p62 in the damaged spinal cord tissues [3,4,68]. Since p62/*Sqstm1* mRNA expression is not changed after SCI, this suggests that an accumulation of autophagosomes after SCI is likely a result of inhibited autophagy flux rather than its increased initiation [1]. This includes diverse injury models such as acute contusion SCI in male rat [4] and male mouse [3] and chronic spinal cord compression in both sexes [66,77]. The inhibition of autophagy flux was also recently reported following moderate contusion SCI in *GFP–LC3* male mice [3], and demonstrated the accumulation of both GFP–LC3 positive autophagosomes and p62 in the same cells near the injury site. Although autophagic changes in response to SCI show some discrepancies observed among studies, species, injury models, and injury severities, the vast majority of data support the inhibition of autophagic flux in the damaged spinal cord.

Increased markers of autophagy, including LC3-II, beclin1, p62, and autophagosomes, have been reported in human TBI autopsy samples [78,79], as well as in cerebrospinal fluid (CSF) [80]. TBI-mediated accumulation of p62 in both brain autopsy samples [78] and CSF [80] is associated with injury severity and worse recovery [80], and likely reflects an impairment of autophagic flux. Similarly, in experimental models of TBI, LC3-II expression levels as well as the accumulation of autophagosomes observed by electron microscope [81] has been demonstrated in the injured brain [79,82,83,84]. However, autophagy flux has not been directly assessed until more recently [62]. Interestingly, these data indicate that unlike in the spinal cord, in the brain, the autophagic flux capacity may be dependent on injury severity [14]. Supporting this possibility, the accumulation of p62 appears most pronounced at or near the lesion area after moderate to severe injury in both rodent TBI models [5,85] and human TBI brain samples [78]. The initiation of autophagy is not changed after moderate/severe injury in a controlled cortical impact (CCI) model [85]. Recently, an inhibition of autophagy flux was also reported in *GFP–LC3* mice after moderate CCI [5], and further confirmed by ex vivo flux assay in organotypic brain slices in the presence or absence of a lysosomal inhibitor (chloroquine). Conversely, in a mild CCI model [85], p62 was reduced in the ipsilateral cortex accompanied by increased beclin 1, ATG5, and ATG12, suggesting the enhancement of autophagy initiation. Increased autophagy has also been reported in rat models of TBI following a fluid percussion injury [83] and CCI [86,87]. Bayir et al. [88] reported that increased LC3-II after moderate CCI injury on postnatal day 17 rats is more prominent in male versus female rats, suggesting that trauma-induced autophagy is not limited to the mature mammalian brain, and that similar to nutrient deprivation studies in vitro [89], there are sex-dependent differences in the autophagic response. The divergent findings in autophagy flux following TBI may be explained by the different lesion paradigms and tissue sampling or time windows after injury. Nonetheless, autophagy flux is impaired in most models of moderate to severe brain injury. 

### 3.3. Lysosomal Functions In Neurotrauma

Lysosomes are membrane-enclosed organelles that contain an array of enzymes that are capable of breaking down all types of biological polymers: proteins, nucleic acids, carbohydrates, and lipids [90,91,92,93]. Lysosomes function as the digestive system of the cell, serving both to degrade material taken up from outside the cell and the obsolete or superfluous components of the cell itself. Autophagic degradation is dependent on lysosomal proteases. Defects in the lysosomal function have been demonstrated in various neurodegenerative diseases and aging. In many of these disease paradigms, lysosomal dysfunction can be caused by the increase in lysosomal membrane permeability (LMP) [94,95,96,97,98,99]. We and others [3,4,5,100] demonstrated that decreased protein levels and activity of lysosomal enzymes accompanied the inhibition of autophagy flux in both TBI and SCI. The altered intracellular localization of soluble lysosomal enzymes including cathepsin D (CTSD) diffuse rather than discrete punctate, which further suggests the possibility that LMP allows the leakage of CTSD into cytosol, resulting in decreased lysosomal activity and the inhibition of autophagy flux after neurotrauma. In addition to its detrimental effect on autophagy, the leakage of the highly degradative lysosomal enzymes into the cytosol could lead to significant damage to other cellular components. 

Similar to most cellular organelles, lysosomes are surrounded by a single-layer phospholipid membrane. Therefore, they are vulnerable to the activation of phospholipases. There are three major phospholipases present in the CNS: namely, calcium-dependent secretory phospholipase A2 (sPLA2), calcium-dependent cytosolic phospholipase A2 (cPLA2), and calcium-independent phospholipase A2 (iPLA2) [101,102]. Among these, cPLA2 is considered to be the most important PLA2 isoform, because it exhibits a strong preference for the deacylation of arachidonic acid (AA) over other fatty acids, and has been implicated as an effector in the receptor-mediated release of AA [103,104]. Both the expression levels and activity of cPLA2 are increased in several neurodegenerative diseases [101,105,106,107,108] as well as following SCI [109,110] and TBI [111]. cPLA2 cleaves the fatty acyl linkage of glycerophospholipids at the sn-2 position, releasing AA and leaving lysophospholipids remaining in the membrane [101,102,112,113,114,115]. Then, AA is oxygenated and further transformed into a variety of products such as prostaglandins, leukotrienes, and thromboxanes, which mediate or modulate inflammatory reactions. The accumulation of lysophospholids can affect the membrane properties, including its fluidity and permeability [112,113,115]. Although the signaling function of cPLA2 metabolites has been widely studied, the consequences of its action on the properties of the affected membranes and organelles are less well understood.

**Figure 1:**
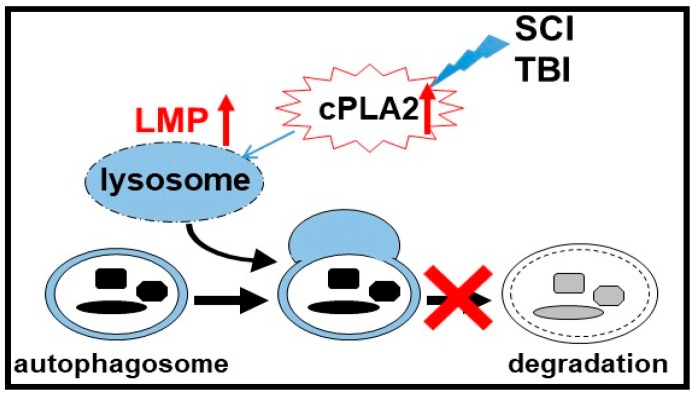
Spinal cord injury (SCI) and traumatic brain injury (TBI) activate cytosolic phospholipase A2 (cPLA2), mediating increased lysosomal membrane permeability (LMP), and leading to lysosomal damage and the inhibition of autophagy after SCI and TBI.

We recently used mass spectrometry (MS)-based in vivo lysosomal lipidomics to demonstrate an increase in several classes of lysosophospholipids, which are the products of phospholipases A (PLAs), as well as the accumulation of PLA activators and ceramides in lysosomes purified from the injured brain [111] and spinal cord [110]. Further in vitro and in vivo data indicated that cPLA2-mediated LMP leads to release of lysosomal enzymes into the cytosol, the inhibition of autophagy flux, and neuronal cell death. Taken together, our data implicate cPLA2 in the mediation of the lysosomal defects observed in the pathophysiology of neurotrauma. cPLA2-mediated lysosomal damage in turn causes the inhibition of autophagy flux and autophagosome accumulation, which are associated with neuronal cell death (Figure 1). Interestingly, our in vitro results indicate that the inhibition of cPLA2 can also limit amyloid-β-induced LMP and the inhibition of autophagy [111], suggesting that similar mechanisms may also contribute to Alzheimer’s and potentially other neurodegenerative diseases.

## 4. Beneficial or Detrimental Effects of Autophagy in CNS Cells After Trauma

### 4.1. Function of Autophagy in Neurons after Neurotrauma

Neurons, the fundamental units of the CNS, are post-mitotic cells, and most neurons have a cell body, an axon, and dendrites. CNS trauma causes progressive secondary injury processes including neuronal cell death and axonal damage, contributing to neurological dysfunction. Neurons are also the cell type that are most commonly associated with dysregulated autophagy after both TBI and SCI [1]. Afterwards, TBI markers of autophagy are predominantly reported in the neurons of the ipsilateral cortex and hippocampus, and appear within 24 h after injury [5,79,83]. In a mouse closed head injury model, Diskin et al. [82] reported that 17–37% of Beclin-1+ neurons were also terminal deoxynucleotidyl transferase dUTP (2’-deoxyuridine 5’-triphosphate) nick end labeling (TUNEL) positive, suggesting that cell death was associated with autophagy. In agreement with this observation, we observed significant numbers of GFP-LC3+ and p62+ neurons expressing cleaved caspase-3 in the injured cortex at day (d) 1 and 3 after injury in a mouse controlled cortical impact CCI model [5]. In addition, neuronal GFP-LC3 and p62 also co-localized with markers of caspase-independent cell death (such as AIF), indicating that impaired autophagy flux may contribute to both apoptotic and non-apoptotic neuronal cell death. 

In SCI, changes in neuronal autophagy appear to be dependent on both the injury model and neuronal subtype. In rat and mouse contusion SCI models, the accumulation of both LC3 and p62 was greater in the ventral horn motor neurons as compared to the dorsal horn sensory neurons, despite the latter being located closer to the impact site [3,4,68]. This suggests that motor neurons may be relatively more vulnerable to the disruption of autophagy flux. Moreover, in these models, motor neurons with impaired autophagy also expressed higher levels of caspase12 and cleaved caspase 3 [4], supporting a role for impaired autophagy in mediating neuronal apoptosis. However, the inhibition of autophagy flux in the dorsal horn neurons was observed in spinal nerve ligation, which is a model of neuropathic pain [116,117], suggesting that neuronal subclasses may respond differentially to specific types of injury. That autophagy flux was inhibited even in this mild injury model also suggests that overall, spinal cord neurons may be more prone to the inhibition of autophagy flux as compared to brain neurons. 

**Figure 2:**
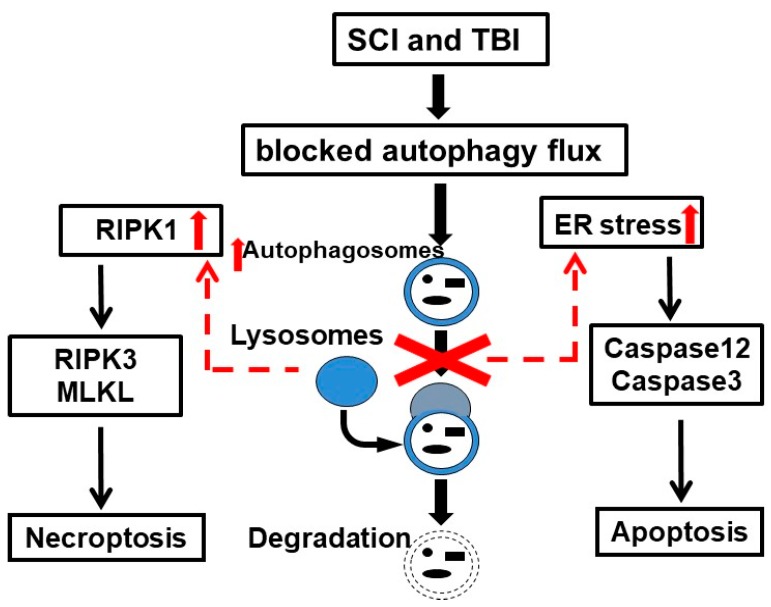
Lysosomal damage after spinal cord injury (SCI) and traumatic brain injury (TBI) cause endoplasmic reticulum (ER) stress and the accumulation of receptor interacting protein kinases (RIPK1/RIPK3), leading to neuronal apoptosis and necroptosis.

Endoplasmic reticulum (ER) stress induces a variety of neuronal cell death pathways, and has been implicated in the secondary injury processes after CNS trauma [118,119], but its mechanisms remain unclear. The induction of ER stress and activation of caspase 12 following TBI and SCI have been previously reported [4,5,118]. ER stress is a potent inducer of autophagy [120,121], and autophagy can protect cells from ER stress-mediated cell death [122]. Consistently, several studies indicate that markers of inhibited autophagy flux after injury correlate with the exacerbation of ER stress, suggesting a connection between the inhibition of autophagy and induction of ER stress-mediated neuronal cell death after neurotrauma [4,5] (Figure 2).

Necroptosis is a programmed form of necrosis, or inflammatory cell death, mediated by the RIPK1/RIPK3 complex [123]. The inhibition of necroptosis can improve functional recovery after both SCI and TBI, suggesting that it is involved in the mediation of the secondary injury [43,44]. A recent study [3] demonstrated the accumulation of markers of necroptosis specifically in neurons displaying signs of autophagy flux inhibition and lysosomal damage, pointing to a previously unexplored link between the inhibition of the autophagy-lysosomal pathway and the induction of neuronal necroptosis after SCI. Additionally, the inhibition of lysosomal function in vitro resulted in the accumulation of necroptosis mediators RIPK1 and RIPK3. Therefore, the inhibition of lysosomal function and autophagy in CNS trauma could also contribute to neuronal necroptosis (Figure 2).

Recent findings have identified another type of cell death called ‘autosis’, which is an autophagy-dependent non-apoptotic form of cell death with unique features. It is characterized by enhanced cell-substrate adhesion, focal ballooning of the perinuclear space, and the dilation and fragmentation of ER. Autosis is mediated by the Na^+^, K^+^-ATPase pump in the mitochondria, and it is triggered by autophagy-inducing peptides, starvation, and neonatal cerebral hypoxia–ischemia. Whether or not autophagy-dependent autosis participates in neuronal cell death in injured CNS is unknown. While it’s known that autophagy can mediate the execution of cell death in the absence of other death pathways, it is difficult to determine in vivo whether cell death is caused by high autophagy or if it is the by-product of other processes that happen alongside autophagy. 

In addition to neuronal cell bodies, autophagosome accumulation has been reported to occur in damaged axons after both TBI and SCI [68,79,124]. While autophagy has been proposed to participate in the process of axonal degeneration in vitro, it is currently not known whether it is involved in axonal damage after neurotrauma. Another possibility is that axonal accumulation of autophagosomes could reflect the inhibition of autophagy flux due to lysosomal defects and cytoskeletal collapse, similar to what is observed in neurodegenerative diseases.

### 4.2. Role of Autophagy in Oligodendrocyte Survival after Neurotrauma

Oligodendrocytes (OLs), also called oligodendroglia, are a type of neuroglia whose main functions are to provide support and insulation to axons, and are the myelinating cells of the CNS [125]. Mature OLs are the end product of oligodendrocyte precursor cells (OPCs), which have to undergo a complex and precisely timed program of proliferation, migration, differentiation, and myelination to finally produce the insulating sheath of axons. Due to this complex differentiation program, and due to their unique metabolism and physiology, OLs count among the most vulnerable cells of the CNS. It is known that OLs undergo apoptosis following CNS trauma, thus the loss and demyelination of OLs contribute as major pathological processes to secondary damages after injury [126]. Increased numbers of autophagosomes have been reported in OLs after both TBI and SCI [4,5,65,66,68,127]. The accumulation of phagophores and autophagosomes in OLs was observed at three to seven days post-injury [4,5,68], which is later than in neurons in most reports. In a mouse contusion SCI model [127], myelin fractions purified from injured spinal cord tissues at eight days post-injury showed enrichment in autophagic proteins, including LC3-II, Atg5, and beclin 1. This study [127] has also demonstrated the increased co-localization of p62 with Olig2 at 3 days post-SCI, indicating impaired autophagic flux in OLs. Additionally, after TBI, increased LC3+ cells have been observed not only in mature OLs, but also in NG2+ OPCs [5].

While the current data strongly support changes in autophagy in OLs during functional recovery after CNS trauma, much less is known about either the mechanisms or the function of autophagy in OL and OPC cells. It has been previously demonstrated in a rat model of demyelination that autophagy is necessary to support oligodendrocyte precursor survival and myelin development [128]. Thus, it is possible that autophagy in OPCs/OLs may play a similar function after CNS trauma. Using transgenic mice with OL-specific loss of the essential autophagy gene *Atg5*, Saraswat et al. reported [127] that the loss of autophagy in OLs exacerbates the inhibition of the autophagic flux in OLs and correlates with worse functional recovery after SCI and greater myelin loss. 

ER stress is also a potent inducer of autophagy in OLs [127]. In vitro, a pharmacological blockade of ER stress-induced autophagy in OPCs increases survival. *Atg5* deletion specifically in OLs increased ER stress and reduced cell viability in vitro and in vivo after SCI, indicating a critical regulation of ER stress by autophagy in OLs. This is consistent with the beneficial contribution of *Atg5* and autophagy to OPC/OL health after SCI. Whether or not *Atg5* and ER stress in OLs/OPCs play a similar function following TBI needs to be determined. 

### 4.3. Function of Autophagy in Microglia and Astrocytes after CNS Trauma

Microglia are a type of glial cell that is located throughout the CNS. They account for 10–15% of all cells found within the brain. As the resident macrophage cells, microglia act as the first and main form of active immune defense in the CNS. Microglia are activated in response to injury and are one of the main drivers of inflammatory responses after both TBI and SCI [129]. An increase in markers of autophagy has been reported in microglia after both TBI and SCI [4,5,66]. Interestingly, only the most activated CD68-expressing microglia with amoeboid morphology were reported to accumulate autophagosomes and p62 after TBI in a mouse CCI model [5]. This is consistent with the recently discovered function for autophagy in the regulation of inflammatory responses in macrophages and other immune cells [130,131], including microglia [132]. In general, high levels of autophagy flux are associated with anti-inflammatory properties and the inhibition of flux, with pro-inflammatory phenotypes [133]. Several autophagy genes are also linked to inflammatory and autoimmune diseases [133,134]. On the mechanistic level, autophagy is thought to control the activity of the inflammasomes, both directly by degrading components of activated NLRP3 (nucleotide-binding oligomerization domain, leucine rich repeat and pyrin domain containing 3) and AIM2 (absent in melanoma 2) inflammasomes, and indirectly by limiting mitochondrial damage and reactive oxygen species (ROS) production [135,136,137]. Additionally, autophagy can modulate inflammatory polarization through its influence on NFκB (nuclear factor kappa B subunit) activity by directly targeting the RELA/p65 protein and by affecting the availability of the p62 protein, which is needed for NFκB activation [138,139,140]. Conversely, a pro-inflammatory environment may also regulate levels of autophagy, as in primary cultured mouse microglia, where interleukin 1 Beta (IL-1β) induced the accumulation of many acidic vesicles loaded with autophagic markers (p62 and LC3) [141]. It is possible that autophagy may similarly be regulated by and contribute to the regulation of inflammatory responses in microglia and/or infiltrating macrophages after CNS injury. 

Astrocytes are the largest and most numerous types of glial cells in the CNS, and are known to have a wide variety of physiological functions, including the maintenance of neurons, formation of the blood–brain barrier, and regulation of synaptic function. Similarly, to microglia, in response to injury, astrocytes are activated and become hypertrophic. Several groups reported that glial fibrillary acid protein (GFAP)-positive astrocytes accumulate autophagosomes after neurotrauma [65,66,82,84]. In mouse contusion SCI, reactive astrocytes accumulated phagophores and autophagosomes at seven d post-injury. Unlike other cell types, this was associated with increased autophagy initiation rather than its inhibition [68]. Much less is known about either the mechanisms or the function of autophagy in astrocytes. Autophagy has been implicated in protective cellular responses in astrocytes [142,143,144]; however, further work will be necessary to determine how it may affect astrocyte survival and function after CNS trauma.

### 4.4. Beneficial or Detrimental Effects of Autophagy Activation in Neurotrauma

Defects in autophagy are thought to contribute to Parkinson’s disease [145], Alzheimer’s disease [146], and other age-related dementias. The accumulated evidence suggests that autophagy deficiency contributes to neurodegenerative diseases by perturbing protein homeostasis, as well as by directly contributing to neuronal cell death, axonal degeneration, and synaptic dysfunction. Conversely, the upregulation of autophagy has been proposed as a potential therapeutic strategy and shows considerable promise in both pre-clinical and clinical studies. However, the function of autophagy in neurodegeneration after traumatic CNS injury has been more controversial [1], with both beneficial and detrimental roles proposed. This may reflect that, as discussed above, either the induction or inhibition of autophagy flux may occur after acute CNS injury, especially in the brain. Since the function of autophagy is dependent on its flux, in cases where flux is inhibited, such as moderate to severe TBI or most cases of SCI, it would be expected to contribute to neuronal cell death and exacerbate other pathological phenotypes. Conversely, in cases where autophagy flux is increased, such as mild TBI, it would be expected to promote cell survival and improve outcomes. Thus, in each neurotrauma model, it is pertinent to ascertain whether observed changes in autophagy are the result of its induction or the inhibition of flux.

## 5. Therapeutic Potential of Autophagy–Lysosomal Pathway Modulation in Neurotrauma

### 5.1. Neuroprotection

The most common drugs used to manipulate autophagy in vivo are the mTOR (mechanistic target of Rapamycin kinase) inhibitor rapamycin, which is used to stimulate autophagy, and the type III PI3 kinase inhibitor 3-methyladenine (3-MA), which inhibits autophagosome formation. In general, pharmacological modulations that promote autophagy flux have been shown to provide neuroprotection in both TBI and SCI. However, in addition to autophagy, rapamycin is known to affect other cellular functions such protein synthesis, cell proliferation, and immune responses. Therefore, it is important to confirm that the neuroprotective effects of rapamycin are mediated via the restoration of the autophagy–lysosomal pathway. In a neonatal hypoxia–ischemia injury model, the protective effects of rapamycin were attenuated in animals treated with 3-MA [147]. Rapamycin-induced neuroprotection was also attenuated by AKT1 inhibition, suggesting that both autophagy and AKT1 signaling may be involved downstream of mTOR. Involvement of this pathway in TBI and SCI remains to be confirmed. However, as an improvement in functional recovery was reported after both pharmacologically enhancing [64,148] or blocking autophagy [149,150] after a thoracic SCI, further studies are warranted.

Another complicated factor is that activity of the target of rapamycin, mTOR, plays a vital role in oligodendrocyte differentiation [151] and myelination, and is also involved in axonal sprouting [152], which are both necessary for functional recovery after CNS injury, especially in the spinal cord. Therefore, the inhibition of mTOR may not be optimal as a treatment for CNS injury in general, and SCI in particular. In agreement with this, some [127,149] reported no effects of rapamycin on functional recovery after SCI. This suggests that drugs that are able to promote autophagy flux without inhibiting mTOR function may offer the best therapeutic benefits after neurotrauma. Trehalose is a naturally occurring sugar containing two glucose molecules, and has been reported as a novel mTOR-independent autophagy enhancer [153,154]. Trehalose has been shown to improve outcomes in rodent models of neurodegenerative diseases [155,156] and in a rabbit model of spinal cord ischemia [157]. However, trehalose is known to also act as a chemical chaperone [153], which could attenuate injury and improve recovery independently of autophagy. A specific mTOR inhibitor, Torin 1 [158,159], has been reported to increase autophagy flux. Both trehalose and Torin 1 induce lysosomal biogenesis via activation of the transcriptional factor E-box (TFEB) [160] and increase autophagy flux but are known to act through different molecular pathways (mTOR-independent and mTOR-dependent, respectively). However, whether or not a combination of trehalose and Torin 1 is able to overcome the block of autophagy flux after neurotrauma and lead to improved functional outcomes needs to be investigated.

As lysosomal function is necessary to support autophagy flux, increasing lysosomal biogenesis has been shown to augment autophagy flux and improve outcomes in animal models of neurodegenerative diseases [161]. Enhancing lysosomal function by reducing lysosomal damage or promoting lysosomal biogenesis may also provide an attractive approach for therapy after neurotrauma. In both contusion TBI and SCI mice models, we recently demonstrated that the early administration of cPLA2 inhibitor arachidonyl trifluoromethyl ketone (AACOCF3) reduced lysosomal damage, improved autophagy flux, limited neuronal cell death, and improved functional outcomes [110,111]. Another possibility is the enhancing activity of the transcription factor EB (TFEB). TFEB is the master regulator of lysosomal biogenesis and is negatively regulated by mTOR [162]. The enhancement of autophagic flux by activating TFEB is protective in experimental brain injuries produced by cadmium [163]. This supports the notion that enhancing autophagy flux by stimulating lysosomal biogenesis may represent a potential treatment strategy after neurotrauma. 

There are also other autophagy inducers reported in the context of neurotruama. Baicalin (7-D-glucuronic acid-5,6-dihydroxyflavone) is a major flavonoid in traditional Chinese medicinal herb isolated from the radix of Scutellaria baicalensis that was found to increase expression levels of LC3, Beclin 1, and p62 at 24 h following the weight-drop TBI model in mice [164]. Salubrinal is the selective phosphatase inhibitor of p-eIF2α. Wang et al. reported [165] that salubrinal reduced the expression of the ER stress marker as well as the number of CHOP+/TUNEL+ and CHOP+/LC3+ cells at 48 h after mouse TBI, which was associated with neuroprotection and improved recovery. Omega-3 polyunsaturated fatty acids (ω-3 PUFA) are known to have anti-oxidative and anti-inflammatory effects. ω-3 PUFA supplementation increased Beclin-1 deacetylation and its nuclear export, and induced direct interactions between cytoplasmic Beclin-1 and Bcl-2 by increasing the sirtuin family of proteins (SIRT1) activity following a weight-drop TBI model in rat [166]. Valproic acid (VPA), a class I/II histone deacetylase inhibitor, is able to significantly increase expression levels of the autophagic markers (LC3-II, Beclin, ATG-3, and ATG-7) at one day after TBI in rat [167]. These data are consistent with the notion that enhancing autophagy may be beneficial after neurotrauma. However, while the treatments affected the expression of autophagy markers after neurotrauma, their influence on autophagy flux remains to be assessed. Additionally, none of the compounds used are specific for autophagy, and have many additional cellular and organismal effects. Therefore, it remains to be determined if their beneficial effects are in fact dependent on autophagy.

### 5.2. Neuroinflammation

The crosstalk between autophagy and inflammation has been reported [168] in diseases associated with inflammation, such as inflammatory bowel diseases [169], type 2 diabetes [170], cardiac disorders [171], and cystic fibrosis [172]. Autophagy is known to be involved in the development, homeostasis, and survival of all inflammatory cells, including macrophages, neutrophils, and lymphocytes, thus playing critical roles in the development and pathogenesis of inflammation. In a mouse Alzheimer’s disease model, a recent study [173] demonstrated positive correlation between Beclin-1, IL-1β, and TNF-α (tumor necrosis factor alpha) in the cortex and/or hippocampus, suggesting a relationship between inflammatory responses and autophagy. It remains to be determined whether a similar relationship exists after neurotrauma, and whether enhancing autophagy can be used to manipulate neuroinflammatory responses in this context. Conversely, as inflammation can affect cellular autophagy [1], the anti-inflammatory treatments could also improve autophagy flux.

Neurotrauma-induced neuroinflammation can cause the excessive generation of reactive oxygen species (ROS), which non-selectively damages neurons and glia, and thus participates in pathophysiology [174]. ROS can also regulate autophagy in both a positive and negative manner, depending on the levels and context [175,176]. Excessive ROS as well as reactive nitrogen species (RNS) can inhibit autophagy through the S-nitrosylation of proteins in the JNK (c-Jun N-terminal kinase) and mTOR pathways, which are important for the regulation of autophagy [177]. Lysosomal membranes can also be directly subjected to oxidative damage, leading to LMP. This suggests that ROS could be an important factor contributing to alterations in autophagy levels after neurotrauma. Conversely, autophagy can also be stimulated by ROS through its influence on ATG4 activity. Therefore, depending on the injury severity and mechanism, CNS injury-mediated ROS and RNS could either stimulate or inhibit autophagy flux.

## 6. Conclusions and Perspectives

When the CNS is injured, many cellular processes are activated that attempt to heal the damage. The activation of other cellular responses can increase damage over time. In case of autophagy, both protective and pathological functions are possible. Although we need to more thoroughly understand the complex and heterogeneous autophagic response triggered by the CNS trauma, in general, the enhancement of autophagy is considered protective in many injury paradigms, and helps restore homeostasis. Autophagy-enhancing drugs could positively affect multiple cell types, promoting neuron and oligodendrocyte survival, oligodendrocyte differentiation, and decreasing neuroinflammation. So, while we need to understand the mechanisms in more detail, the enhancement of autophagy after TBI and SCI carries a promise of a potential pleiotropic treatment that could target multiple cell types and pathways.
